# Electric field-induced rupture kinetics of giant unilamellar vesicles with varying gramicidin A content in membrane

**DOI:** 10.1371/journal.pone.0338817

**Published:** 2026-01-05

**Authors:** Md. Tariqul Islam Bhuiyan, Mir Jubair Ahamed, Rajia Sultana, Tawfika Nasrin, Md. Kabir Ahamed, Md. Masum Billah, Mohammad Abu Sayem Karal

**Affiliations:** 1 Department of Physics, Bangladesh University of Engineering and Technology, Dhaka, Bangladesh; 2 Department of Physics, Fareast International University, Dhaka, Bangladesh; 3 Department of Physics, Bangladesh University of Textiles, Dhaka, Bangladesh; 4 Radiation, Transport and Waste Safety Division, Bangladesh Atomic Energy Regulatory Authority, Dhaka, Bangladesh; 5 Department of Physics, Jashore University of Science and Technology, Jashore, Bangladesh; University at Buffalo, UNITED STATES OF AMERICA

## Abstract

In this study, we examine the rupture kinetics of giant unilamellar vesicles (GUVs) composed of DOPG/DOPC and varying mole fractions of gramicidin A (GrA), under externally applied electric fields. Time-resolved fluorescence microscopy reveals that the presence of GrA modulates vesicle rupture behavior in a non-monotonic fashion. At lower contents of GrA (0–0.05 mole%), GUVs exhibit enhanced structural resilience, with significantly reduced rupture probability, suggesting increased membrane stability. However, at higher contents of GrA (0.05–5%), vesicles display accelerated rupture kinetics, reflecting decreased membrane integrity. Quantitative analysis of rupture events indicates a biphasic dependence of the rupture rate constant on GrA content. The pore-edge tension was obtained from the slope of the fitted linear relation between the logarithm of the rupture rate constant and the inverse of effective membrane tension for different GrA%. The pore-edge tension varies nonlinearly with GrA%, reaching a minimum at intermediate GrA levels (0.05%). These findings are consistent with a theoretical model for electroporation, where pore formation is governed by a balance between destabilizing electric tension and stabilizing line tension. The results highlight how lower mole% of GrA enhance membrane mechanical robustness, while excessive GrA incorporation disrupts bilayer cohesion and accelerates electroporation. This study provides new insights into the tunable electromechanical properties of lipid membranes modulated by membrane-active peptides.

## 1. Introduction

Membrane proteins play essential roles in various biological processes that are essential to cell survival and function. These proteins mediate the transport of ions and larger solutes across membranes and facilitate cellular communication through receptor interactions [1]. One commonly employed peptide in membrane studies is Gramicidin A (GrA), which generates membrane potential by allowing ion flux across lipid bilayers [2,3]. Due to its small size and ease of chemical modification, GrA serves as an excellent model for studying the properties of real ion channels, as they share similar structural characteristics [4]. Here, the “similar structural characteristics” refer to the shared features between GrA and naturally occurring ion channels—specifically, their ability to form transmembrane pores, exhibit selective ion transport, and undergo conformational changes in response to environmental conditions. Ion channels regulate ionic permeability [5] and control ion transport in excitable cell membranes [6]. Previous studies have shown that GrA plays a crucial role in various cellular processes, including T-cell signaling [7], antimicrobial peptide-induced pore formation, and the entry of cell-penetrating peptides [8,9]. Additionally, it has been observed that an increase in the binding constant between peptides and membranes significantly enhances poration kinetics [10].

Irreversible electroporation (IRE) is an advanced technique widely utilized for tissue and cancer cell ablation [11,12]. This method applies a high-intensity electric field to cells, inducing the formation of membrane pores and drastically increasing membrane permeability. IRE has gained recognition for its potential biomedical applications, particularly in cancer treatment [13]. However, studying the structural and functional alterations in cell membranes is complex due to their heterogeneous composition and dynamic properties [14]. As a result, researchers often rely on simplified models, such as giant unilamellar vesicles (GUVs), which closely resemble cell membranes in size and behavior. GUVs provide a well-controlled experimental platform for real-time observations under an optical microscope, offering valuable insights into membrane biophysics [15,16].

Membrane tension is a fundamental physical property influencing various cellular processes. Proper regulation of membrane tension is crucial for maintaining cellular integrity and function [17,18]. In biological systems, osmotically induced membrane tension plays a key role in triggering electropermeabilization in living cells [19,20]. When an external force—such as electrical, mechanical, or optical tension—induces pore formation in a lipid bilayer, it generates pore-edge tension (Γ) within the membrane [21–24]. This parameter, also referred to as line tension, determines the stability and closure of membrane pores. It is intrinsically linked to membrane stability, resealing dynamics, and the energy required to sustain an open pore. An increase in monolayer spontaneous curvature due to tension-induced pore formation results in a lower Γ, ultimately enhancing vesicle rupture kinetics [25].

When an external electric field is applied, it induces lateral electric tension (*σ*_e_) in vesicle membranes. If it surpasses a critical threshold, membrane poration occurs, leading to either transient permeability changes or complete rupture. Similarly, mechanical tension can be applied via micropipette aspiration, enabling precise control over membrane mechanics [26]. Recent studies have explored the effects of lipid composition, surface charge density, cholesterol concentration, and osmotic pressure on vesicle rupture kinetics in the absence of GrA [27]. However, incorporation of GrA in the membranes plays a significant role in modulating electroporation responses. Understanding its influence on electric field-induced rupture is crucial for understanding cellular behavior under physiological and pathological conditions.

Our recent study [28] focused on mechanical stress–induced deformation and rupture of GUVs, whereas the present study investigates electric field–induced rupture kinetics. The novelty of the current work lies in the quantitative analysis of electroporation dynamics modulated by varying mole fractions of GrA, including the determination of rupture rate constants and pore-edge tensions as a function of both GrA content and membrane tension. This provides new kinetic and energetic parameters that were not addressed in our previous mechanical stress study. Moreover, electric-field-induced rupture represents a distinct physicochemical regime compared to mechanical compression, involving field-driven charge redistribution, dielectric breakdown, and electro-compressive tension, which interact with GrA differently than mechanical deformation. By systematically varying both GrA% and membrane tension, we reveal a biphasic electromechanical response that highlights how low vs. high GrA content modulates pore nucleation and expansion under electric stress.

Therefore, the primary objective of this research is to investigate the electric field-induced rupture kinetics of GUVs and quantify pore-edge tension in membranes containing various mole fractions of GrA. By examining electroporation regimes in the presence of GrA, this study aims to provide a comprehensive understanding of membrane behavior under electrical stress. The findings will contribute valuable insights into electroporation mechanisms and aid in the development of electroporation-based biomedical applications.

## 2. Materials and methods

### 2.1. Chemicals and reagents

1,2-dioleoyl-*sn*-glycero-3-phospho-(1´-*rac*-glycerol) (sodium salt) (DOPG) and 1,2-dioleoyl-*sn*-glycero-3-phosphocholine (DOPC) were purchased from Avanti Polar Lipids Inc. (Alabaster, AL). Besides, gramicidin A (GrA) (produced by *Bacillus brevis* and extracted from its culture), 1,4-piperazinediethanesulfonic acid (PIPES), bovine serum albumin (BSA), O,O´-bis (2-aminoethyl) ethyleneglycol-*N*,*N*,*N*´,*N*´-tetraacetic acid (EGTA), calcein, sodium chloride (NaCl), glucose, and sucrose were obtained from Sigma-Aldrich (Germany).

### 2.2. Preparation of GUVs under physiological conditions

Giant unilamellar vesicles (GUVs) with DOPG/DOPC compositions and varying mole fractions of GrA (e.g., 0, 0.01, 0.05, 0.1, 1, 3, 5) were synthesized using the natural swelling method [29]. The membranes of the GUVs were composed of varying molar ratios of DOPG, DOPC, and GrA, such as DOPG/DOPC/GrA (40/60/0), (40/59.99/0.01), (40/59.95/0.05), (40/59.9/0.1), (40/59/1), (40/57/3), and (40/55/5). To synthesize GUVs with different GrA%, DOPG and DOPC lipids (dissolved in chloroform) and GrA (dissolved in ethanol) were mixed gently in a glass vial, with a total volume of 200 μL. The components were allowed to mix naturally due to diffusion, and the vial was gently shaken by hand to ensure homogeneity. The mixture was left undisturbed for 10 minutes. Another vial was prepared in the same manner to ensure consistency across experiments. Following this, the solvents were evaporated under a gentle nitrogen flow, and the vials were placed in a vacuum desiccator connected to a rotary vacuum pump for at least 12 hours to ensure complete drying. Subsequently, 20 µL of MilliQ water was introduced into each vial, and the samples were pre-hydrated by incubating at ~ 48°C for 8 minutes in a mini water bath. After pre-hydration, 1 mL of buffer (10 mM PIPES, 150 mM NaCl, pH 7.0, and 1 mM EGTA) containing 0.10 M sucrose was added to each vial, and the samples were incubated at 37°C for 2.5 hours to facilitate vesicle formation. The choice of temperatures in the GUV preparation method was based on optimizing different stages of vesicle formation. The pre-hydration step was carried out at ~ 48°C to enhance lipid mobility and promote uniform swelling of the dry lipid film, as elevated temperatures above the lipid phase transition temperature facilitate membrane hydration and lamellar separation. The subsequent incubation step for vesicle growth was conducted at 37°C to mimic physiological conditions and ensure that the formed GUVs were in a fluid phase suitable for later electroporation experiments. To separate GUVs from any formed aggregates, the samples underwent centrifugation at 13,000 × *g* (where *g* represents gravitational acceleration) at 20°C for 20 minutes using a centrifuge (NF 800R Centrifuge, Nuve, Turkey). After the gentle centrifugation step, the supernatant was carefully collected for subsequent experiments, as it contains the intact GUVs, while the pellet mostly comprises lipid aggregates and multilamellar vesicles. In contrast to our previous protocol using phase-contrast microscopy to visualize GUVs in the region of uniform electric field between the anode and cathode, we encountered optical disturbances in the present experiments. Specifically, in some trials, bubble formation—likely due to electrode surface corrosion—occurred near the electrodes during field application. These bubbles occasionally interfered with the optical path and compromised the quality of phase-contrast images. To circumvent this limitation, we employed fluorescence microscopy by incorporating a fluorescent probe into the GUV interior, which allowed unambiguous vesicle visualization despite the occasional presence of bubbles. This modification ensured consistent image quality and reliable rupture time determination across all experimental conditions. In case of preparing the fluorescent probe (calcein) encapsulated GUVs, vesicles were produced in a solution with 1 mM calcein and 0.10 M sucrose in buffer. The resulting GUV suspension was further purified using a membrane filtration technique [30–32]. The purified GUVs were then transferred to a fresh buffer solution containing 0.10 M glucose. GUV dynamics were examined using a phase-contrast fluorescence microscope (IX 73 Olympus, Japan) equipped with a 20 × objective. The observations were carried out at 25 ± 1°C, maintained by a Tokai Hit Thermo Plate (Japan). A charge-coupled device (CCD) camera (DP22, Olympus) was used to capture images of the GUVs at a frame rate of 25 frames per second.

### 2.3. Electric field-induced membrane tension in GUVs

Following the transfer of purified GUV suspension into a microchamber, an external electric field (*E*) was applied to induce electro-permeabilization. When GUV was exposed to an electric field, a transmembrane voltage (*V*_m_) developed across the lipid bilayer, leading to the generation of lateral membrane tension (*σ*_e_) within the membrane. This tension can be mathematically expressed as [33]:


$$\sigma_{\text{e}}=\varepsilon_{\text{m}}\varepsilon_{\text{0}}\left(\frac{h}{\text{2}h_{\text{e}}^{\text{2}}}\right)V_{\text{m}}^{\text{2}}$$ (1)

where *ε*_m_ is the membrane permittivity (approximately 4.5), *ε*_0_ is the permittivity of free space, *h* represents the membrane thickness (~ 4 nm), *h*_*e*_ is the membrane dielectric thickness (~ 2.8 nm). Eq (1) follows from electro-mechanical coupling principles, where the stored electrostatic energy per unit area in the membrane is proportional to its effective capacitance and the square of *V*ₘ. Since the experimented GUVs are typically spherical, the angle (*θ*) between the applied electric field direction and the normal to the bilayer surface varies across the vesicle’s membrane, ranging from 0° to 90°. The transmembrane voltage (*V*_m_) at a given *θ* is given by: *V*_m_ = 1.5*RE*|cosθ|, where *R* is the radius of the vesicle. The highest value of *V*_m_ occurs when *θ*** = **0°, leading to *V*_m_ = 1.5 *RE*. The expression *V*_m_ = 1.5*RE*|cos*θ*| is derived from the analytical solution of Laplace’s equation for a spherical vesicle placed in a uniform external electric field, as originally described by Schwan [34] and further developed by others [35–37]. This formulation assumes: (i) the vesicle behaves as a thin dielectric shell with uniform thickness *h*, (ii) the conductivity of the inner and outer aqueous media is much greater than that of the membrane, and (iii) the field is applied in the low-frequency (DC or quasi-static) limit. In such conditions, the potential difference across the membrane is maximal at the poles (*θ *= 0° and 180°), where *V*_m_ = 1.5*RE*, and decreases with ∣ cos*θ* ∣ toward the equator (*θ* = 90°). This critical voltage, denoted as *V*_c_, determines the threshold for vesicle rupture. By substituting *V*_m_ into Eq. (1), an expression for lateral membrane tension is obtained [38]:


$$\sigma_{\text{e}}=\text{2}\text{2}.\text{8}\text{6}\;R^{\text{2}}E^{\text{2}}\;(\text{mN}/\text{m})$$ (2)

where *R* is the radius of the GUV (m), *E* is the magnitude of applied electric field (V/m). The rupture threshold is influenced by both vesicle size and the intensity of the applied electric field. For instance, if *R* = 10 μm and *E* = 553 V/cm, the resulting transmembrane voltage is *V*_m_** = **0.83 V, while the induced lateral membrane tension is *σ*ₑ **= **7 mN/m.

In the investigations, the choice of *σ*ₑ = 8 mN/m was motivated by the need to probe rupture kinetics within a physiologically relevant and experimentally accessible membrane tension range. In electroporation theory, the induced membrane tension influences both the lifetime of metastable prepores and the rate of pore expansion. Tensions in the range of 6–10 mN/m are sufficiently high to induce pore formation on experimentally observable time scales (sec), but not so high as to cause immediate catastrophic rupture that would prevent kinetic analysis [38]. Suppose, at tensions significantly above 10 mN/m, vesicles rupture almost instantaneously, preventing reliable determination of rate constants and pore-edge tension. Therefore, membrane tension *σ*ₑ = 8 mN/m was chosen as a representative, controlled condition that allows robust statistical comparison across different GrA%, enabling the effective study of rupture kinetics of GUVs with varying GrA content in the membrane. When a ‘single GUV’ is placed in an external electric field, dielectric polarization occurs due to the permittivity contrast between the lipid bilayer and the surrounding aqueous medium. This polarization leads to the development of a transmembrane potential (*V*ₘ), which is not uniform but varies with the angle *θ* relative to the field direction. The induced *V*ₘ causes the accumulation of charges at the membrane–water interface, giving rise to an electrocompressive Maxwell stress. This stress translates into an in-plane (lateral) membrane tension (*σ*ₑ) that acts to stretch the membrane.

### 2.4. Experimental setup for applying the electric field to GUVs

**Fig 1:**
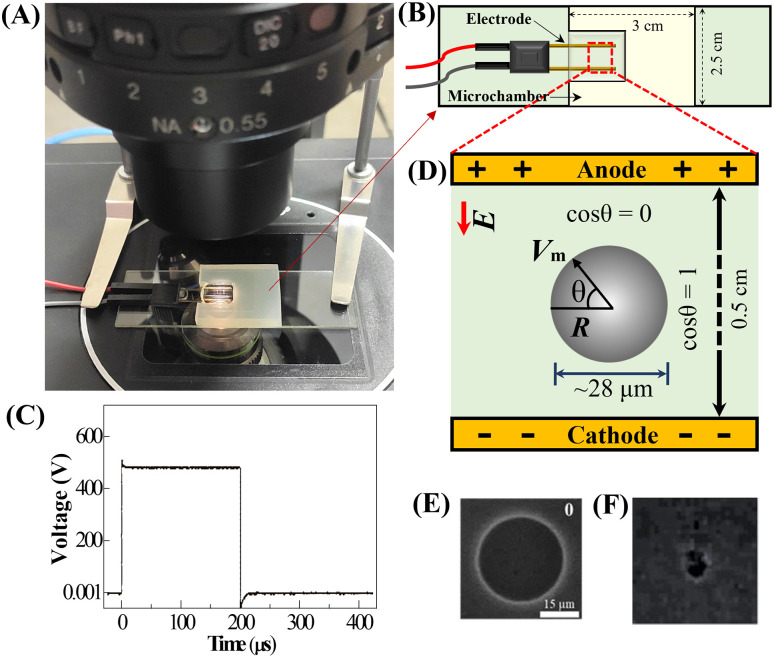
Experimental setup for applying an electric field to GUV. **(A)** Laboratory setup showing the microchamber positioned on the microscope stage during experiments. **(B)** Schematic illustration of the microchamber and the arrangement of gold-coated electrodes. The microchamber was constructed by placing a U-shaped silicone rubber spacer on a glass slide to maintain structural integrity. **(C)** Graph depicting the applied pulsating DC signal with a frequency of 1.1 kHz over time (ON time 200 μs). **(D)** Diagram representing a single GUV suspended between the gold-coated electrodes, where *E* denotes the applied electric field. The transmembrane potential (*V*_m_) is dependent on the angle (*θ*) relative to the electric field direction. **(E)** Phase-contrast microscope image of an intact GUV before exposure to the electric field. **(F)** Phase-contrast image showing the same GUV after undergoing rupture due to applied electric field.

To investigate the impact of an external electric field on an individual GUV, experiments were conducted using vesicles (containing different mole fractions of GrA in membrane) suspended in solutions. The schematic representation of the experimental setup utilized for applying the electric field is depicted in Fig 1(A), while the microchamber dimensions and electrode configuration are illustrated in Fig 1(B). A pulsating DC signal with a frequency of 1.1 kHz was employed throughout the study (Fig 1C). Since vesicle suspensions naturally contain GUVs of varying sizes, each experiment was performed on a ‘single GUV’ carefully selected from the suspension having nearly same contrast and diameter. The chosen GUVs for analysis had diameters ranging between 28–32 μm. To ensure statistical reliability, multiple trials were conducted by examining many individual GUVs using several microchambers. Prior to applying the electric field, the diameter of each selected ‘single GUV’ was measured. Using this measurement, the required membrane tension was determined based on Eq. (2). The same technique was utilized in our several investigations [27,38–44]. For each GrA concentration, at least 12–18 (*N* = 12–18) individual GUVs were examined and analyzed in each independent trial (*n* = 2–4) under identical conditions.

**Fig 2:**
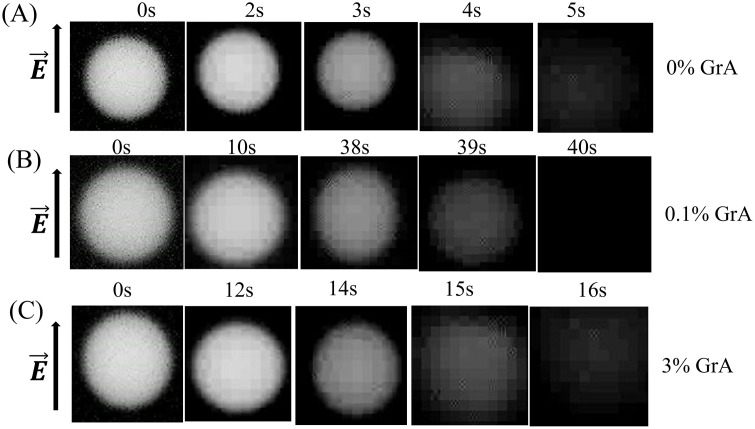
Electric field-induced rupture of DOPG/DOPC/GrA-GUVs at different GrA% under *σ*_e_ = 8 mN/m. Typical fluorescence microscopic images illustrate the rupture progression of **(A)** DOPG/DOPC (40/60)-GUVs, **(B)** DOPG/DOPC/GrA (40/59.9/0.1)-GUVs, and **(C)** DOPG/DOPC/GrA (40/57/3)-GUVs. The applied electric field (*E*) is directed as indicated by the arrow on the left. The time in seconds after applying the electric field is noted in each image.

The corresponding electric field strength (*E*) applied to the vesicles ranged between 250–450 V/cm. The membrane tension (*σ*_e_) was maintained for a maximum duration of 60 s during each experiment. Fig 2(D) provides a schematic representation of an individual GUV positioned between two gold-coated electrodes. The moment at which the vesicle underwent complete rupture was recorded as the onset of pore formation. A representative image of a GUV before the application of the electric field is presented in Fig 1(E), while Fig 1(F) illustrates the same GUV after rupture.

## 3. Results and discussion

### 3.1. Effects of GrA% on the electroporation of GUVs

To investigate the influence of GrA on the electroporation process in DOPG/DOPC/GrA-GUVs, we conducted a series of experiments to examine the rupture dynamics under an applied electric field. Fig 2 presents representative experimental outcomes for GUVs rupture at *σ*_e_ = 8 mN/m, comparing vesicles with 0, 0.1, and 3 mole% GrA in their membranes. At the start of the application of electric field (i.e., at *t* = 0 s), the considered GUV remained their original spherical and intact structures as observed in Fig 2(A). At 0% GrA, the GUV remained almost stable up to 3 s, after which it was ruptured at 4 s, evidenced by the disappearance of its structural integrity. In contrast, for 0.1% GrA, the vesicle remained almost intact for a significantly longer duration (38 s) before rupturing at 39 s (Fig 2B). A different trend was observed at 3% GrA, where the rupture was occurred at 15 s (Fig 2C). Noteworthy, the rupture time and rate constant were obtained from time-lapse image sequences (1 s/frame) for each vesicle. Data are presented as mean ± standard deviation (SD).

The breakdown of vesicles is associated with the formation and growth of pores in the membrane under the applied electric field. The rupture initiation time corresponds to the onset of detectable membrane destabilization, marking the loss of GUV structural stability. During this process, a gradual decrease in lumen fluorescence intensity of the encapsulated calcein dye was observed prior to complete rupture. This fluorescence decrease likely reflects an early stage of membrane permeation or localized leakage, followed by a rapid loss of intensity as the vesicle disintegrates. The accompanying minor shape fluctuations observed just before rupture suggest transient perturbations of the membrane but do not allow us to directly resolve the underlying molecular mechanism. Therefore, while the observed fluorescence dynamics are consistent with previously proposed models involving nanopore formation and subsequent membrane disintegration [45], the present experiments do not directly test this mechanism. The onset of rupture was operationally defined as the time point corresponding to the pronounced drop in lumen fluorescence intensity.

In our experiments, membrane rupture was identified by a pronounced and irreversible decrease in the luminal fluorescence intensity of calcein dye encapsulated within the GUVs. This decrease directly reflects the loss of membrane integrity and the consequent leakage of the fluorescent probe into the external medium. Such a correlation between fluorescence decay and membrane rupture has been well established in previous peptide–membrane interaction and electroporation studies [46,47]. We previously demonstrated that the sudden drop in internal fluorescence corresponds to vesicle rupture or large pore formation [48]. We employed the same criterion and confirmed that the fluorescence decrease coincides with the morphological collapse of GUVs, as captured by time-lapse microscopy. Therefore, the sharp decline in luminal fluorescence intensity in the present study reliably indicates physical membrane rupture.

The imaging time resolution of 1 s/frame and the spatial resolution corresponding to a 15 μm scale bar were selected to appropriately capture the mesoscale (intermediate scale — between the microscopic (molecular) and macroscopic (bulk) levels.) dynamics of vesicle deformation and rupture, rather than the nanoscopic events of individual channel opening. The open time of a single GrA channel typically lies in the microsecond to millisecond range, and the pore diameter (at initial stage) is on the order of few nanometers—well below the optical resolution limit of conventional microscopy. In contrast, the formation and expansion of electroporation-induced pores in GUVs occur over hundreds of milliseconds to several seconds, and at spatial scales from submicron to tens of microns. Therefore, a temporal resolution of 1 s/frame provides sufficient sampling to resolve the global morphological transitions (e.g., swelling, rupture, and membrane retraction) without excessive photo-bleaching or data redundancy. The chosen optical magnification and scale (15 μm) ensure the full vesicle contour remains in the field of view while preserving adequate pixel resolution for quantitative image analysis of rupture kinetics. Thus, the selected temporal and spatial resolutions were optimized for the characteristic spatiotemporal scales relevant to GUV electroporation and peptide-induced membrane destabilization, rather than for direct observation of transient molecular channel events.

### 3.2. Stochastic nature of GUV rupture and dependence on GrA concentration

**Fig 3:**
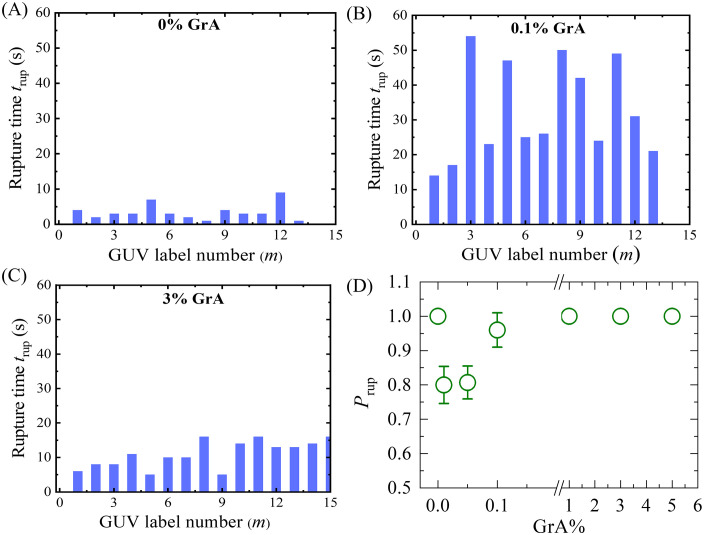
Analysis of rupture time (*t*_rup_) of DOPG/DOPC/GrA-GUVs at different GrA% under *σ*_e_ = 8 mN/m. **(A–C)** Stochastic rupture behavior of many ‘single GUVs’ in one independent experiment at (A) 0% GrA, (B) 0.1% GrA, and (C) 3% GrA. The x-axis represents the GUV label number **(*m*)**. **(D)** Probability of rupture (*P*_rup_) as a function of GrA%, showing an initial decrease followed by an increase with higher GrA%.

To ensure statistical reliability, multiple independent experiments were conducted, where 12–18 GUVs (*N *= 12–18) were analyzed per experimental run (*n* = 3–4 independent experiments) under identical conditions at *σ*_e_ = 8 mN/m. The rupture time (*t*_rup_) exhibited variability among individual GUVs within an independent experiment, indicating a stochastic rupture behavior that depends on the GrA%, as illustrated in Fig 3(A, B, C). A comparative analysis of rupture times revealed that GUVs with 0% GrA ruptured faster compared to those with 0.1% GrA. Interestingly, GUVs containing 3% GrA ruptured more quickly than those with 0.1% GrA, suggesting a non-monotonic dependence of rupture time on GrA content. The rupture time is defined as the duration required for the complete disintegration of a GUV under the applied electric field (when the GUV’s lumen-intensity decreased remarkably). Each ‘single GUV’ was monitored for up to 60 s, regardless of whether rupture occurred or not. A similar rupture trend was observed for vesicles containing other GrA content (0.01%, 0.05%, 1%, and 5%) at *σ*_e _= 8 mN/m. The probability of rupture *P*_rup_, which is defined as the ratio of the number of ruptured GUVs to the total number of GUVs analyzed under identical conditions as a function of GrA% is depicted in Fig 3(D). The data reveal that *P*_rup_ initially decreases with increasing GrA%, but beyond a certain threshold, it begins to rise, indicating that higher GrA levels may promote membrane instability beyond a critical point.

### 3.3. Rate constant of rupture in DOPG/DOPC/GrA-GUVs with varying GrA% at fixed membrane tension

**Fig 4:**
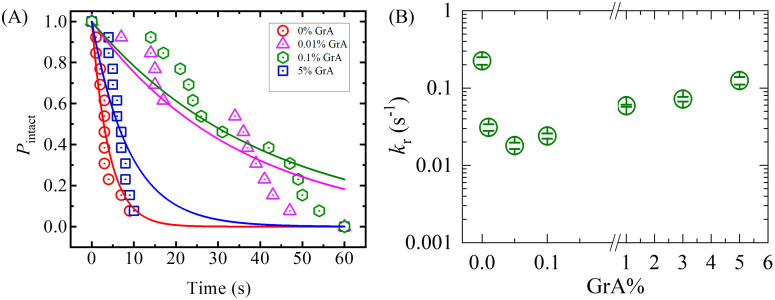
Rupture kinetics of DOPG/DOPC/GrA-GUVs at different GrA% under *σ*_e_ = 8 mN/m. **(A)** Time-dependent fraction of intact GUVs (*P*_intact_) for 0, 0.01, 0.05, and 5% GrA, showing the exponential decay behavior. The coefficient of determination (*R^2^*) was evaluated for the goodness of fit shown in **(A)**. The values of *R^2^* were obtained 0.90, 0.91, 0.91, and 0.79 for 0, 0.01, 0.05, and 5% GrA, respectively. **(B)** GrA%-dependent variation in *k*_r_, highlighting a non-monotonic trend in rupture susceptibility. The error bars in (B) represent the standard error of the mean rate constants derived from 3–4 independent experiments for each GrA%.


To quantify the rupture kinetics of GUVs at different GrA% while maintaining a constant membrane tension (*σ*_e_), we analyzed the time-dependent fraction of intact vesicles (*P*_intact_) that remained unruptured throughout the observation period. This fraction represents the fraction of GUVs that retained their structure over time and is mathematically expressed as:


$$P_{\text{intact}}(t)=\;\text{1}\;-\;P_{rup}$$ (3)

where *P*_rup_ denotes the cumulative probability of rupture at a given time *t*. For instance, if a total of 18 GUVs were examined at *σ*_e_ = 8 mN/m, and 9 GUVs ruptured within 60 s, the corresponding *P*_intact_ would be: *P*_intact_ = 1 − *P*_rup_ = 1 − 9/18 = 0.5. Fig 4(A) illustrates the experimental time course of *P*_intact_ for different GrA% at 8 mN/m. The data reveal that the decay in *P*_intact_ varies as GrA% increases from 0% to 5% in the membrane, suggesting a GrA-dependent rupture process. The decay behavior follows a single-exponential function, given by:

$$P_{\text{intact}}(t)=\text{exp}\left(-k_{r}t\right)$$ (4)

where *t* represents the time duration for which the membrane tension is applied. The experimentally fitted decay curves allowed for the determination of the rupture rate constant (*k*_r_) at different GrA%. For a single typical experiment, the extracted values of *k*_r_ for 0%, 0.01%, 0.05%, and 5% GrA were 24.3 × 10 ⁻ ^2^ s ⁻ ¹, 2.8 × 10 ⁻ ^2^ s ⁻ ¹, 1.9 × 10 ⁻ ^2^ s ⁻ ¹, and 11.5 × 10 ⁻ ^2^ s ⁻ ¹, respectively (Fig 4A). Fig 4(A) illustrates the experimental time course of *P*_intact_ for different GrA% at 8 mN/m. The time dependent *P*_intact_ for 0.1, 1, and 3% GrA is provided in the Supporting Information (S1 File). To ensure the robustness of our findings, multiple independent trials were conducted. The averaged values of *k*_r_ obtained for 0, 0.01, 0.05, 0.1, 1, 3, and 5% GrA at *σ*_e_ = 8 mN/m were (22.5 ± 1.40)×10 ⁻ ^2^ s ⁻ ¹, (3.1 ± 0.19)×10 ⁻ ^2^ s ⁻ ¹, (1.8 ± 0.10)×10 ⁻ ^2^ s ⁻ ¹, (2.4 ± 0.14)×10 ⁻ ^2^ s ⁻ ¹, (3.4 ± 0.18)×10 ⁻ ^2^ s ⁻ ¹, (7.2 ± 0.30)×10 ⁻ ^2^ s ⁻ ¹, and (12.5 ± 0.9)×10 ⁻ ^2^ s ⁻ ¹, respectively for *N* = 12–18 and *n* = 2–4. Thus, *k_r_* decreases sharply from 22.5 × 10 ⁻ ^2^ s ⁻ ¹ at 0% GrA to a minimum of 1.8 × 10 ⁻ ^2^ s ⁻ ¹ at 0.05% GrA, before rising again to 12.5 × 10 ⁻ ^2^ s ⁻ ¹ at 5% GrA, demonstrating a clear nonmonotonic dependence on GrA content. Thus, the *k*_r_ values reveal a nonlinear dependence on GrA%. Initially, the rupture rate constant decreases as GrA content increases, reaching a minimum at 0.05% GrA. However, beyond this point, a further increase in GrA content leads to a progressive increase in *k*_r_, indicating an enhanced susceptibility of the vesicle membrane to rupture (Fig 4B). These findings suggest that at lower GrA%, the membrane peptide may stabilize the membrane, reducing rupture likelihood. However, at higher contents, the presence of GrA may facilitate membrane destabilization, possibly by altering bilayer mechanical properties or increasing membrane permeability, thus accelerating rupture kinetics.

### 3.4. Rate constant of rupture in DOPG/DOPC/GrA-GUVs under varying GrA% and membrane tensions

**Fig 5:**
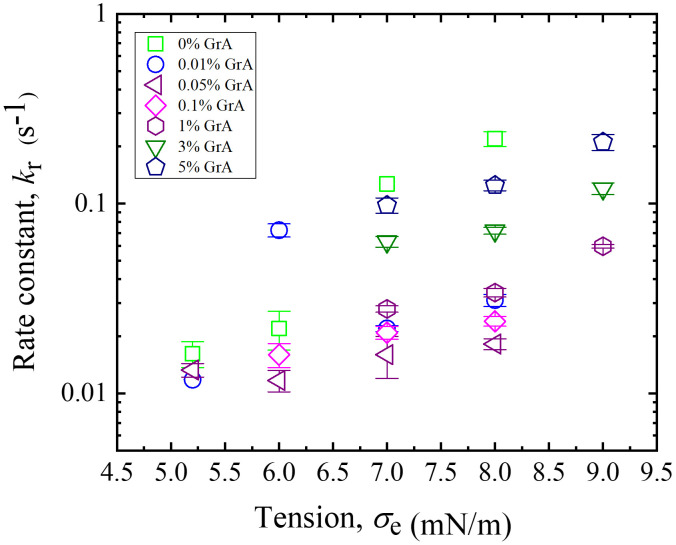
Rate constant of rupture of DOPG/DOPC/GrA-GUVs at different GrA% and electric tensions. Different symbols indicate distinct GrA% in the membranes. Error bars represent standard deviations.

This section presents the analysis of membrane tension (*σ*_e_)-induced rupture in DOPG/DOPC/GrA-GUVs under varying GrA% and membrane tensions. The results are summarized in Fig 5. The same experimental approach described in Section 3.3 was employed to evaluate the rupture rate constant (*k*_r_) for varying GrA% and different values of *σ*_e_. The mean values of *k*_r_ for DOPG/DOPC/GrA-GUVs are displayed in Fig 5, illustrating a clear increase in *k*_r_ with higher electric tension across all GrA%. Moreover, at a constant *σ*_e_, the presence of GrA significantly affects the rupture kinetics of vesicles, suggesting that GrA incorporation alters membrane stability and influences the rupture process. The trend shows that *k*_r_ increases as *σ*_e_ rises across all GrA%. At a fixed *σ*_e_, *k*_r_ exhibits a biphasic dependence on GrA%, initially decreasing with lower GrA% and then increasing at higher GrA levels. Error bars represent standard errors, indicating variations from multiple independent measurements. As shown in Fig 5, the rupture rate constant (*k*_r_) increases with higher membrane tension (*σ*_e_) because elevated tension lowers the energy barrier for pore formation, making it more likely for prepores to expand into irreversible transmembrane pores. Physically, higher *σ*_e_ contributes an additional destabilizing term in the pore free energy, which accelerates the rupture process.

### 3.5. Estimation of pore-edge tension (Γ) in DOPG/DOPC/GrA-GUVs under different GrA%

**Fig 6:**
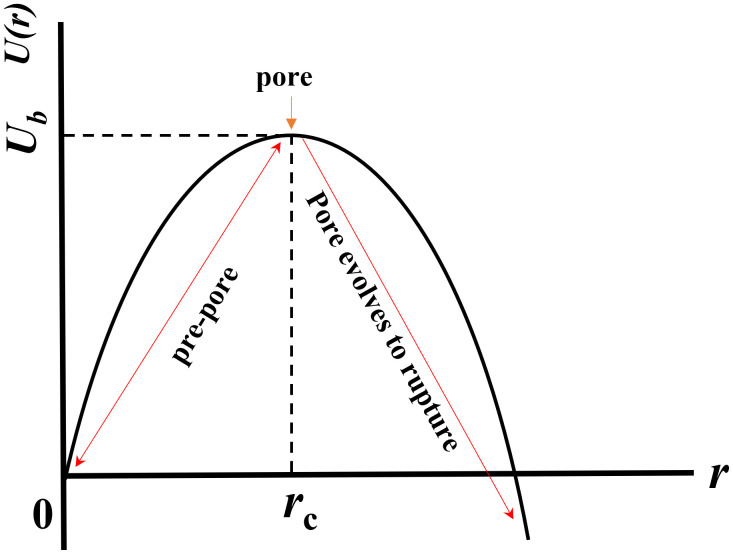
The energy landscape of a system undergoing conformational changes in the process of pore formation. This consists of a prepore region and the rupture region. The curve illustrates the transition from a stable prepore state to an unstable pore state at the energy barrier *U*_b_, followed by progression toward membrane rupture. The critical pore radius *r*_c_ marks the transition point between reversible pore formation and irreversible rupture.

Lipid bilayers, composed of amphiphilic lipid molecules, continuously undergo spontaneous fluctuations in lateral packing density. These fluctuations create localized regions of lower lipid density, often referred to as prepores or transient density rarefactions [35,49]. When subjected to an external electric field (*E*), the membrane experiences an induced lateral tension (*σ*_e_), which promotes pore formation. Additionally, thermal energy contributes to fluctuations in lipid density, leading to localized condensation and expansion within the bilayer [50]. If a rarefied region surpasses a critical radius (*r*_c_), it evolves into a prepore with a characteristic radius (*r*). When *r* < *r*_c_, the prepore rapidly closes. Conversely, when *r *≥ *r*_c_, the prepore transitions into a transmembrane pore, eventually leading to vesicle rupture. These dynamic events, including the transition from prepore to full membrane rupture, are schematically illustrated in Fig 6. Vesicle rupture occurs within ~1 s as the radius of the prepore expands indefinitely. It refers to the timescale of the irreversible transition from a critical prepore to full membrane rupture under the applied membrane tension. Specifically, once a prepore reaches the critical radius *r*_c_, it becomes energetically favorable for the pore to expand without bound, leading to vesicle rupture. The process is extremely rapid, occurring on the order of ~1 s, and does not imply that the pore remains open for 1 s. Instead, this duration reflects the transient period between the prepore surpassing *r*_c_ (critical radius), and the complete collapse of the vesicle.

The free energy associated with a prepore, *U*(*r*, *σ*_e_), consists of two opposing terms: (1) A destabilizing term (-π*r*^*2*^*σ*_e_), which promotes pore expansion by reducing the energy barrier. (2) A stabilizing term (2π*r*Γ), which resists pore expansion and accounts for the pore-edge tension (Γ), defined as the free energy per unit length of the prepore boundary. Following classical pore formation theory, the free energy expression for a prepore is given by [51]:


$$U\left(r,\sigma_{\text{e}}\right)=\;2\pi r\Gamma -\;\pi (\sigma_{\text{e}}+\;B)r^{\text{2}}\;$$ (5)

where *B* represents the component of membrane tension-induced by electrostatic interaction due to membrane surface charge. Based on prior findings [51], *B* is estimated to be 1.76 mN/m, as the surface charge density and ionic conditions of DOPG/DOPC/GrA-GUVs in buffer match those of DOPG/DOPC (40/60)-GUVs. The activation energy barrier (*U*_b_) for pore formation corresponds to the maximum free energy value at *r* = *r*_c_, expressed as [51]:


$$U_{\text{b}}=\frac{{\pi \Gamma }^{\text{2}}}{\left(\sigma_{\text{e}}\;+\;B\right)}$$ (6)

From Eq. (6), the Arrhenius relationship for the rate constant (*k*_r_) can be derived as [52]:


$$k_{\text{r}}=A\;\text{exp}\left[-\frac{\frac{\pi \Gamma^{\text{2}}}{{(\sigma }_{\text{e}}+B)}}{k_{\text{B}}\text{T}}\right]$$ (7)

where, *A* is the frequency factor, *k*_B_ is the Boltzmann constant, and *T* is the absolute temperature. By rearranging Eq (7), we can derive the revised expression as follows:


$$\text{ln}k_{\text{r}}=\text{ln}A-\frac{\pi \Gamma^{\text{2}}}{k_{\text{B}}\text{T}}\left(\frac{\text{1}}{{(\sigma }_{\text{e}}+B)}\right)=C-\frac{\pi \Gamma^{\text{2}}}{k_{\text{B}}\text{T}}\left(\frac{\text{1}}{{(\sigma }_{\text{e}}+B)}\right)\;\;\;\;\;\;\;$$ (8)

**Fig 7:**
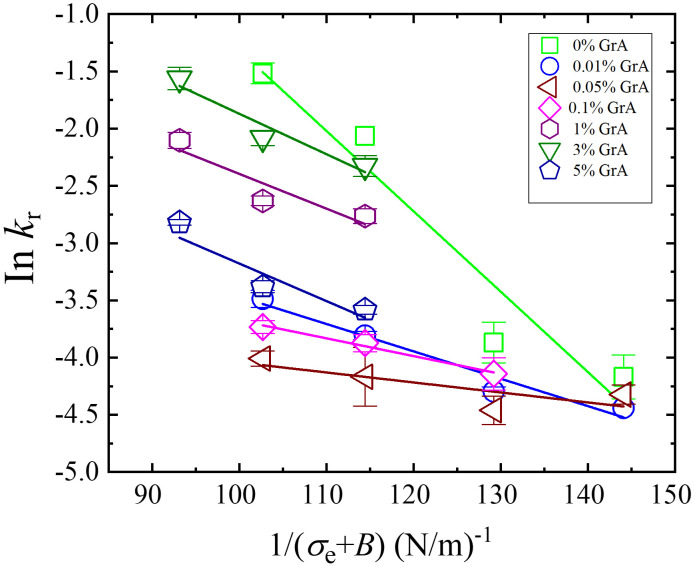
Dependence of ln*k*_r_ on 1/(*σ*_e_* *+ *B*) for DOPG/DOPC/GrA-GUVs at various GrA%. Each symbol represents a different GrA%, as indicated in the legend. The solid lines represent best-fit theoretical curves based on Eq (8), demonstrating the relationship between membrane tension and rupture kinetics across different GrA conditions.

Experimental data for ln*k*_r_ plotted against 1/(*σ*_e _+ *B*) for different GrA% were fitted to Eq. (8), as shown in Fig 7. The best-fit analysis provided the estimated values of pore-edge tension (Γ) for different GrA%: Γ = 9.6 ± 0.5, 5.6 ± 0.3, 3.4 ± 0.2, 4.5 ± 0.2, 6.5 ± 0.3, 6.3 ± 0.3, 6.8 ± 0.3 pN for 0, 0.01, 0.05, 0.1, 1, 3, and 5% GrA, respectively. These values align well with previous results obtained for DOPG/DOPC (40/60)-GUVs in buffer and are comparable to those reported for DOPG/DOPC/GrA (40/59.99/0.01)-GUVs [53].

### 3.6. Impact of GrA% on rupture kinetics and membrane stability

Table 1 summarizes the dependence of key parameters—including the rate constant of rupture, probability of rupture, and pore-edge tension—on the mole fraction of GrA in the membrane under *σ*_e_ = 8 mN/m. The data reveal a biphasic trend in the variation of these parameters. At lower GrA contents (0–0.05%), a decrease in rate constant, probability of rupture, and pore-edge tension suggests an increase in membrane mechanical stability. This is likely due to GrA-induced changes in lipid packing and lateral interactions, leading to a more rigid bilayer. However, between 0.05%

**Table 1:** Rate constant, probability of rupture, and pore-edge tension in DOPG/DOPC/GrA-GUVs with varying GrA% under *σ*_e_ = 8 mN/m.

GrA mole%	Rate constant, *k*_r_ (s^-1^)	Probability of rupture (at *t* = 60s), *P*_rup_	Pore-edge tension, (pN)
0	(22.5 ± 1.40)×10^−2^	1 ± 0	9.6 ± 0.5
0.01	(3.1 ± 0.19)×10^−2^	0.80 ± 0.05	5.6 ± 0.3
0.05	(1.8 ± 0.10)×10^−2^	0.80 ± 0.05	3.4 ± 0.2
0.1	(2.4 ± 0.14)×10^−2^	0.96 ± 0.05	4.5 ± 0.2
1	(3.4 ± 0.18)×10^−2^	1 ± 0	6.5 ± 0.3
3	(7.2 ± 0.30)×10^−2^	1 ± 0	6.3 ± 0.3
5	(12.5 ± 0.9)×10^−2^	1 ± 0	6.8 ± 0.3

Noteworthy, the probability of rupture ( *P*rup) reported in Table 1 represents the cumulative probability of rupture at *t* = 60 s, i.e., the fraction of GUVs that have ruptured within 60 s under the applied membrane tension (*σ*e= 8 mN/m). This value provides a direct measure of the membrane’s stability at a given GrA concentration.

and 1% GrA, these parameters mostly start to rise again, indicating a reduction in membrane stability. This softening effect at higher GrA contents may result from various physical factors, including excessive ion channel formation, which disrupts lipid organization, increases bilayer fluidity, and weakens membrane integrity.

## 4. General discussion

### 4.1. Mechanistic insights into electric field-induced pore formation (rupture occurred due to pore formation) in DOPG / DOPC / GrA - GUVs by varying GrA %

The present study elucidates the impact of GrA incorporation into lipid bilayer on the electric field-induced rupture kinetics of DOPG/DOPC/GrA-GUVs under physiological conditions. The experimental results consistently demonstrate a non-monotonic dependence of rupture susceptibility on GrA%, reflecting complex biophysical interactions between membrane mechanics, peptide-induced perturbations, and stochastic pore formation processes. The electric field induces lateral membrane tension (*σ*ₑ), modulating the free energy landscape of the bilayer and promoting pore nucleation and growth [35]. The observed rupture behavior as a function of GrA content reveals a biphasic trend: (1) At lower GrA contents (0–0.05%), a substantial reduction in rupture rate constant (*k*_r_) and rupture probability (*P*_rup_) was observed. This suggests that trace amounts of GrA stabilize the membrane, possibly by: enhancing lipid packing via lateral ordering effects [54], altering membrane compressibility, and inhibiting pore expansion. At higher GrA contents (0.05–5%), *k*_r_ and *P*_rup_ increase gradually. This could result from: GrA-induced thinning of the membrane and increased permeability, enhanced dielectric breakdown susceptibility due to ion channel formation, increased curvature stress or lateral heterogeneity, promoting pore nucleation [55,56]. This non-monotonic response underscores a critical concentration threshold beyond which GrA compromises membrane integrity rather than reinforcing it.

To describe the GrA-dependent pore-edge tension Γ(*ϕ*), we now introduce the mole fraction of GrA in the bilayer (*ϕ*) which is defined as the ratio of mole number of GrA in the bilayer to the sum of mole number of phospholipids and mole number of GrA in the membrane. The biphasic behavior suggests a non-monotonic dependence of Γ on GrA content in membrane as follows:


$$\Gamma (\varphi )=\;\;\Gamma_{\text{0}}\;+a\emptyset \;-b\emptyset^{\text{2}}\;$$ (9)

where Γ_0_ is the pore-edge tension of the pure lipid bilayer in the absence of GrA, *a* is coefficient representing initial membrane stiffening due to GrA (ordering effect), and *b* is the coefficient representing membrane softening or curvature induction at high *ϕ*. This parabolic dependence captures the experimental trend: pore edge tension decreases initially, reaches a minimum, and then increases with further GrA enrichment. In Eq. (9) the quadratic term was chosen because our experimental results show that Γ(*ϕ*) decreases with increasing *ϕ* up to a certain GrA mole fraction, after which it increases again, suggesting a competition between two mechanisms: (1) Destabilization at low–moderate *ϕ* due to lipid disordering and hydrophilic exposure at the rim. (2) Re-stabilization at higher *ϕ* due to possible GrA aggregation, formation of ordered rim-associated peptide domains, or partial shielding of rim hydrophobic exposure.

Substituting Γ(*ϕ*) into the energy barrier equation (Eq. 6) and then into the rupture rate equation (Eq. 7), we obtain the rupture rate constant as a function of GrA content as follows:


$$k_{\text{r}}=A\text{exp}\left[-\frac{\pi }{k_{\text{B}}\text{T}}.\frac{{\left(\Gamma_{\text{0}}\;+\;a\varphi \;-b\varphi^{\text{2}}\right)}^{\text{2}}}{{(\sigma }_{\text{e}}+B)}\right]$$ (10)

This equation explicitly links GrA content with rupture rate and accounts for the biphasic kinetic profile observed experimentally (described in results section). This model integrates the membrane mechanics framework with molecular-level interactions induced by GrA incorporation. At lower GrA levels, *aϕ* dominates, lowering Γ and thus the rupture kinetics, consistent with GrA-induced membrane thinning, hydrophobic mismatch, and increased curvature propensity. As *ϕ* increases further, the − *bϕ*^2^ term becomes significant, capturing a stabilizing effect—possibly due to GrA clustering, bilayer stiffening, or formation of more ordered domains. This leads to increase Γ and rate constant. The resulting non-monotonic rupture rate reflects the balance between line tension reduction and steric saturation effects, providing a theoretical explanation for the biphasic kinetics observed in the experiments. This behavior is analogous to previous studies of peptide-lipid interactions where membrane-active agents modulate bilayer rupture through combined mechanical and thermodynamic influences [54–56].

It should be noted here, under classical pore nucleation theory, a decrease in pore-edge tension (Γ) is generally expected to lower the energy barrier for pore formation and hence increase the rupture rate constant (*k*_r_). However, our data for low GrA mole fractions (0–0.05%) suggest that additional factors influence membrane rupture in this regime. At very low GrA contents, we hypothesize that GrA incorporation induces subtle changes in lipid packing and hydration at the pore rim, which could increase local bending rigidity (*κ*) and/or reduce the effective line tension for pore expansion despite a nominal decrease in the measured Γ. These changes may also alter the initial defect formation probability, such that the nucleation step becomes rate-limiting rather than pore growth. In other words, the decrease in Γ measured under steady conditions may not directly translate into a higher dynamic rupture probability if the pore nucleation barrier is dominated by local lipid-peptide interactions at these low peptide densities. A related possibility is that the lower Γ reflects an average property of the bilayer under tension, but the actual rupture pathway at low GrA content may require cooperative rearrangements of lipids and peptides that are kinetically slower. This could explain the observed decrease in *k*_r_ and *P*_rup_ despite reduced Γ.

**Fig 8:**
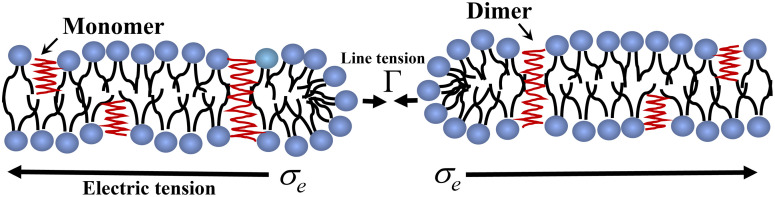
Schematic representation of GrA incorporation and electromechanical response of the lipid bilayer under an applied electric field. GrA monomers insert perpendicularly into each lipid leaflet and can dimerize across the bilayer to form ion-conducting channels spanning the membrane. Upon exposure to an external electric field, electric tension (*σ*ₑ) acts laterally on the bilayer, promoting local deformation and pore initiation. The balance between the applied *σ*ₑ and intrinsic line tension (Γ) governs the stability of the pore edge. Depending on the GrA content and field strength, these perturbations can facilitate membrane thinning and transient pore formation, providing a mechanistic basis for electroporation behavior in peptide-doped membranes.

Fig 8 illustrates a conceptual model for the integration of GrA into lipid bilayers and the hypothesized transition to toroidal pore formation under electric stress. This schematic underpins several mechanistic interpretations relevant to the non-monotonic rupture kinetics observed in our experiments. The bilayer scheme shows GrA molecules existing as monomers and dimers embedded within the bilayer. GrA dimers approximately span the bilayer thickness to form cation-selective channels [55], facilitating ion transport. At low molar fractions (0–0.05%), GrA insertion is thought to stabilize the bilayer by promoting lateral ordering and suppressing fluctuations, thereby increasing the energetic barrier for spontaneous pore formation. At higher GrA fractions (0.05–5%), the increased density of GrA dimers may lead to local curvature stress, membrane thinning, and reduced dielectric thickness, which collectively predispose the membrane to electroporation-induced rupture. This is supported by the report that elevated GrA concentrations disrupt lipid packing and increase membrane susceptibility to destabilization [56].

The electric field-induced tension and pore nucleation are illustrated. The thermodynamic free energy of a pre-pore is given by eq (5). As GrA content increases, its influence on Γ becomes non-trivial. Our estimated values of Γ from lnk_r_ versus 1/(*σ*_e_ + *B*) analysis suggest that GrA initially enhances Γ (via membrane ordering), then reduces it beyond a threshold, facilitating pore growth and rupture. Toroidal pore (shown in Fig 8) differs from classical hydrophobic pores by having lipid headgroups lining the pore wall, stabilizing the high-curvature geometry [57]. In the case of GrA-induced pore formation, the toroidal pore configuration is energetically favorable because the lipid headgroups bend inward to line the pore, reducing the exposure of hydrophobic tails to water and thereby lowering the overall free energy.

GrA has been proposed to facilitate such curvature transitions, especially at high contents. The local hydrophobic mismatch and membrane thinning can promote elastic deformations that favor toroidal over cylindrical pore structures [58]. This mechanistic insight aligns with our observation that higher GrA fractions increase rupture probability and rate constants, possibly due to toroidal pore nucleation at GrA-rich microdomains. At higher GrA content, formation of toroidal pores becomes favorable due to enhanced membrane curvature stress and local thinning. The bending energy per unit area of such a curved structure is given by the classical Helfrich bending energy model [59]:


$$E_{\text{bend}}=\frac{\text{1}}{\text{2}}\kappa {\left(C-C_{\text{0}}\right)}^{\text{2}}$$ (11)

where *κ* is the bending rigidity of the membrane, *C* is the actual local curvature of the membrane, *C*_0_ is the spontaneous curvature induced by embedded molecules like GrA [59–61]. GrA, due to its asymmetric insertion, hydrophobic mismatch, or local perturbation of lipid packing, can increase *C*_0_ in specific domains, thereby reducing the local bending energy and promoting toroidal pore formation [60–62]. The presence of GrA can locally modulate membrane curvature and decrease bending rigidity, facilitating the formation of these high-curvature toroidal pores. Consequently, the local bending energy contribution stabilizes the pore structure, making it more favorable than a classical hydrophobic pore under similar tension. However, an increase in the spontaneous curvature (*C₀*) may not universally lead to a reduction in local bending energy; the outcome depends on the relationship between *C* (the actual curvature of the membrane) and *C₀*, as described by the Helfrich bending energy model. Specifically, when *C* is close to *C₀*, the bending energy is minimized, facilitating the formation of curved structures such as toroidal pores. In contrast, if the induced *C₀* is far from the prevailing membrane curvature, the bending energy may increase instead. In the context of GrA, its asymmetric insertion, hydrophobic mismatch, and local lipid packing perturbations can induce local *C₀* that is comparable to the curvature required for pore formation, thereby lowering the energetic barrier for these events.

In lipid bilayer enriched with GrA, the membrane becomes softer and more prone to curvature instabilities. When $C\approx C_{\text{0}}(\varphi ),\;$with $C_{\text{0}}(\varphi )\propto \;\varphi ,$ where *ϕ* is the mole fraction of GrA in membrane, the membrane curvature matches the induced spontaneous curvature, thereby minimizing the energy cost of forming highly curved structures like toroidal pores. These GrA-rich domains thus serve as nucleation hotspots, explaining the observed increase in rupture probability at higher GrA% [63]. The hypothesis presented in Fig. (8) provides a compelling framework for understanding the biphasic effects of GrA on electroporation. At lower contents, GrA acts as a membrane stabilizer, while at higher mole fraction, it becomes a destabilizing agent promoting curvature-driven defects and toroidal pore formation. This model reconciles the observed kinetic trends with molecular-level phenomena and extends previous findings on GrA-membrane interactions.

### 4.2. Biophysical implications and outlook

Our results provide key insights into how membrane-active peptides such as GrA modulate lipid membrane stability under electric stress. This dual modulation—stabilization at low mole fractions and destabilization at higher concentrations—has several important implications: (i) Optimizing electroporation-based drug delivery: Understanding the concentration-dependent effects of GrA can inform the design of liposome or vesicle carriers that leverage transient membrane permeabilization for controlled cargo release. (ii) Peptide-induced cytotoxicity: The mechanistic insights into GrA’s influence on membrane rupture may help interpret its role in antibiotic activity, channel formation, and selective cytotoxic effects. (iii) Designing synthetic vesicles and vesicle engineering: Our findings offer guidance for engineering vesicles with tunable mechanical robustness and controlled susceptibility to rupture, which is relevant for biomimetic systems and synthetic biology applications. Future studies combining continuum mechanics modeling and molecular dynamics simulations will be valuable to dissect the molecular underpinnings of GrA’s biphasic effects, offering predictive capabilities for peptide-lipid interactions under stress. While the present DOPG/DOPC system provides a controlled platform to understand the effect of GrA, future studies incorporating more complex lipid and protein compositions will be essential to capture the full biophysical diversity of cell membranes relevant to IRE-based cancer therapies.

## 5. Conclusions

In this study, we systematically investigated how varying mole fractions of gramicidin A (GrA) influence the rupture kinetics in DOPG/DOPC/GrA-GUVs under externally applied electric fields. Our results demonstrate that GrA incorporation modulates the electromechanical stability of lipid membranes in a non-monotonic manner. At lower contents (0–0.05%) GrA appears to stabilize the membrane, delaying rupture and significantly reducing the rupture rate constant and rupture probability. This stabilizing effect is attributed to the interaction of GrA with lipid molecules, which may enhance membrane cohesion and increase the energy barrier for pore formation. However, at higher contents (>0.05%), GrA destabilizes the membrane, as evidenced by increased rate constant and probability of rupture, suggesting that beyond a critical threshold, GrA facilitates membrane rupture. This behavior likely arises from GrA-induced alterations in bilayer mechanical properties and local membrane perturbations that promote pore nucleation and expansion. The dependence of rupture kinetics on GrA was consistently observed across different electric field-induced membrane tensions, with all data showing a biphasic trend in rupture behavior. Furthermore, the analysis of the pore-edge tension and energy barrier confirmed the dual role of GrA. These findings are quantitatively supported by a thermodynamic model of pore formation, which aligns well with the experimental data and highlights how membrane-active peptides like GrA can finely tune membrane mechanical responses. Overall, our work provides new insights into the mechanistic underpinnings of peptide–lipid interactions during electroporation, revealing how small variations in peptide concentration can profoundly affect membrane resilience. While the present study utilizes GUVs as a simplified model system, extending these investigations to nanometer-sized vesicles and other classes of membrane-active peptides will be crucial to generalize the observed electromechanical principles toward realistic drug delivery and biomedical applications.

## Supporting information

S1 File Electric field-induced rupture kinetics of giant unilamellar vesicles with varying gramicidin A content in membrane. (PDF)

## References

[1] Cournia Z, Allen TW, Andricioaei I, Antonny B, Baum D, Brannigan G, et al. Membrane protein structure, function, and dynamics: a perspective from experiments and theory. J Membr Biol. 2015;248(4):611–40. doi: 10.1007/s00232-015-9802-0 26063070 PMC4515176

[2] Bamberg E, Läuger P. Channel formation kinetics of gramicidin A in lipid bilayer membranes. J Membr Biol. 1973;11(2):177–94. doi: 10.1007/BF01869820 4131309

[3] Lundbaek JA, Collingwood SA, Ingólfsson HI, Kapoor R, Andersen OS. Lipid bilayer regulation of membrane protein function: gramicidin channels as molecular force probes. J R Soc Interface. 2010;7(44):373–95. doi: 10.1098/rsif.2009.0443 19940001 PMC2842803

[4] Shen J, Liu G, Han Y, Jin W. Artificial channels for confined mass transport at the sub-nanometre scale. Nat Rev Mater. 2021;6(4):294–312. doi: 10.1038/s41578-020-00268-7

[5] Kulbacka J, Choromańska A, Rossowska J, Weżgowiec J, Saczko J, Rols M-P. Cell Membrane Transport Mechanisms: Ion Channels and Electrical Properties of Cell Membranes. Adv Anat Embryol Cell Biol. 2017;227:39–58. doi: 10.1007/978-3-319-56895-9_3 28980039

[6] Flood E, Boiteux C, Lev B, Vorobyov I, Allen TW. Atomistic simulations of membrane ion channel conduction, gating, and modulation. Chem Rev. 2019;119(13):7737–832. doi: 10.1021/acs.chemrev.8b00630 31246417

[7] Ma Y, Poole K, Goyette J, Gaus K. Introducing membrane charge and membrane potential to T cell signaling. Front Immunol. 2017;8:1513. doi: 10.3389/fimmu.2017.01513 29170669 PMC5684113

[8] Moghal MMR, Islam MZ, Hossain F, Saha SK, Yamazaki M. Role of membrane potential on entry of cell-penetrating peptide transportan 10 into single vesicles. Biophys J. 2020;118(1):57–69. doi: 10.1016/j.bpj.2019.11.012 31810658 PMC6950768

[9] Moghal MMR, Hossain F, Yamazaki M. Action of antimicrobial peptides and cell-penetrating peptides on membrane potential revealed by the single GUV method. Biophys Rev. 2020;12(2):339–48. doi: 10.1007/s12551-020-00662-z 32152921 PMC7242587

[10] Rashid MMO, Moghal MMR, Billah MM, Hasan M, Yamazaki M. Effect of membrane potential on pore formation by the antimicrobial peptide magainin 2 in lipid bilayers. Biochim Biophys Acta Biomembr. 2020;1862(10):183381. doi: 10.1016/j.bbamem.2020.183381 32504547

[11] Miller L, Leor J, Rubinsky B. Cancer cells ablation with irreversible electroporation. Technol Cancer Res Treat. 2005;4(6):699–705. doi: 10.1177/153303460500400615 16292891

[12] Al-Sakere B, André F, Bernat C, Connault E, Opolon P, Davalos RV, et al. Tumor ablation with irreversible electroporation. PLoS One. 2007;2(11):e1135. doi: 10.1371/journal.pone.0001135 17989772 PMC2065844

[13] Belehradek M, Domenge C, Luboinski B, Orlowski S, Belehradek J, Mir LM. Electrochemotherapy, a new antitumor treatment. First clinical phase I-II trial. Cancer. 1993;72(12):3694–700. doi: 10.1002/1097-0142(19931215)72:12<3694::aid-cncr2820721222>3.0.co;2-2 7504576

[14] Israelachvili JN. Electrostatic forces between surfaces in liquids. Intermolecular and Surface Forces. Elsevier. 2011. p. 291–340. doi: 10.1016/b978-0-12-375182-9.10014-4

[15] Dimova R. Giant vesicles and their use in assays for assessing membrane phase state, curvature, mechanics, and electrical properties. Annu Rev Biophys. 2019;48:93–119. doi: 10.1146/annurev-biophys-052118-115342 30811220

[16] Dimova R, Marques CM. The giant vesicle book. CRC Press, Taylor & Francis Group. 2019.

[17] Keren K. Membrane tension leads the way. Proc Natl Acad Sci U S A. 2011;108(35):14379–80. doi: 10.1073/pnas.1111671108 21873200 PMC3167539

[18] Pontes B, Monzo P, Gauthier NC. Membrane tension: A challenging but universal physical parameter in cell biology. Seminars in Cell & Developmental Biol. 2017;71:30–41. doi: 10.1016/j.semcdb.2017.08.030 28851599

[19] Barrau C, Teissié J, Gabriel B. Osmotically induced membrane tension facilitates the triggering of living cell electropermeabilization. Bioelectrochemistry. 2004;63(1–2):327–32. doi: 10.1016/j.bioelechem.2003.11.009 15110297

[20] Zong W, Li Q, Zhang X, Han X. Deformation of giant unilamellar vesicles under osmotic stress. Colloids Surf B Biointerfaces. 2018;172:459–63. doi: 10.1016/j.colsurfb.2018.08.053 30196231

[21] Brochard-Wyart F, de Gennes PG, Sandre O. Transient pores in stretched vesicles: role of leak-out. Physica A: Statistical Mechanics and its Applications. 2000;278(1–2):32–51. doi: 10.1016/s0378-4371(99)00559-2

[22] Karatekin E, Sandre O, Guitouni H, Borghi N, Puech P-H, Brochard-Wyart F. Cascades of transient pores in giant vesicles: line tension and transport. Biophys J. 2003;84(3):1734–49. doi: 10.1016/S0006-3495(03)74981-9 12609875 PMC1302742

[23] Levadny V, Tsuboi T, Belaya M, Yamazaki M. Rate constant of tension-induced pore formation in lipid membranes. Langmuir. 2013;29(12):3848–52. doi: 10.1021/la304662p 23472875

[24] Leomil FSC, Zoccoler M, Dimova R, Riske KA. PoET: automated approach for measuring pore edge tension in giant unilamellar vesicles. Bioinform Adv. 2021;1(1):vbab037. doi: 10.1093/bioadv/vbab037 36700098 PMC9710609

[25] Tazawa K, Yamazaki M. Effect of monolayer spontaneous curvature on constant tension-induced pore formation in lipid bilayers. J Chem Phys. 2023;158(8):081101. doi: 10.1063/5.0135561 36859073

[26] Evans E, Smith BA. Kinetics of Hole Nucleation in Biomembrane Rupture. New J Phys. 2011;13:095010. doi: 10.1088/1367-2630/13/9/095010 21966242 PMC3182099

[27] Karal MAS, Ahamed MK, Ahmed M, Mahbub ZB. Recent developments in the kinetics of ruptures of giant vesicles under constant tension. RSC Adv. 2021;11(47):29598–619. doi: 10.1039/d1ra04647k 35479542 PMC9040846

[28] Ahamed MJ, Nasrin T, Rakhy ZT, Billah MM, Karal MAS. Effect of gramicidin A on the constant tension-induced rupture of giant unilamellar vesicles and the underlying mechanisms. Chem Phys Lipids. 2025;271:105525. doi: 10.1016/j.chemphyslip.2025.105525 40633828

[29] Reeves JP, Dowben RM. Formation and properties of thin-walled phospholipid vesicles. J Cell Physiol. 1969;73(1):49–60. doi: 10.1002/jcp.1040730108 5765779

[30] Tamba Y, Terashima H, Yamazaki M. A membrane filtering method for the purification of giant unilamellar vesicles. Chem Phys Lipids. 2011;164(5):351–8. doi: 10.1016/j.chemphyslip.2011.04.003 21524642

[31] Karal MAS, Rahman M, Ahamed MK, Shibly SUA, Ahmed M, Shakil MM. Low cost non-electromechanical technique for the purification of giant unilamellar vesicles. Eur Biophys J. 2019;48(4):349–59. doi: 10.1007/s00249-019-01363-6 30918998

[32] Karal MAS, Nasrin T, Ahmed M, Ahamed MK, Ahammed S, Akter S, et al. A new purification technique to obtain specific size distribution of giant lipid vesicles using dual filtration. PLoS One. 2021;16(7):e0254930. doi: 10.1371/journal.pone.0254930 34324548 PMC8321220

[33] Dimova R, Bezlyepkina N, Jordö MD, Knorr RL, Riske KA, Staykova M, et al. Vesicles in electric fields: Some novel aspects of membrane behavior. Soft Matter. 2009;5(17):3201. doi: 10.1039/b901963d

[34] Schwan HP. Electrical properties of tissue and cell suspensions. Adv Biol Med Phys. 1957;5:147–209. doi: 10.1016/b978-1-4832-3111-2.50008-0 13520431

[35] Kotnik T, Rems L, Tarek M, Miklavčič D. Membrane electroporation and electropermeabilization: mechanisms and models. Annu Rev Biophys. 2019;48:63–91. doi: 10.1146/annurev-biophys-052118-115451 30786231

[36] Dimitrov DS. Electroporation and Electrofusion of Membranes. Handbook of Biological Physics. Elsevier. 1995. p. 851–901. doi: 10.1016/s1383-8121(06)80011-4

[37] Angelova MI, Dimitrov DS. Liposome electroformation. Faraday Discuss Chem Soc. 1986;81:303. doi: 10.1039/dc9868100303

[38] Ahamed MK, Karal MAS, Ahmed M, Ahammed S. Kinetics of irreversible pore formation under constant electrical tension in giant unilamellar vesicles. Eur Biophys J. 2020;49(5):371–81. doi: 10.1007/s00249-020-01440-1 32494845

[39] Karal MAS, Ahamed MK, Rahman M, Ahmed M, Shakil MM, Siddique-E-Rabbani K. Effects of electrically-induced constant tension on giant unilamellar vesicles using irreversible electroporation. Eur Biophys J. 2019;48(8):731–41. doi: 10.1007/s00249-019-01398-9 31552440

[40] Ahamed MK, Ahmed M, Karal MAS. Quantification of pulsed electric field for the rupture of giant vesicles with various surface charges, cholesterols and osmotic pressures. PLoS One. 2022;17(1):e0262555. doi: 10.1371/journal.pone.0262555 35025973 PMC8757908

[41] Karal MAS, Ahamed MK, Mokta NA, Ahmed M, Ahammed S. Influence of cholesterol on electroporation in lipid membranes of giant vesicles. Eur Biophys J. 2020;49(5):361–70. doi: 10.1007/s00249-020-01443-y 32535676

[42] Karal MAS, Ahamed MK, Orchi US, Towhiduzzaman M, Ahmed M, Ahammed S, et al. An investigation into the critical tension of electroporation in anionic lipid vesicles. Eur Biophys J. 2021;50(1):99–106. doi: 10.1007/s00249-020-01477-2 33245397

[43] Sarkar MK, Karal MAS, Ahmed M, Ahamed MK, Ahammed S, Sharmin S, et al. Effects of osmotic pressure on the irreversible electroporation in giant lipid vesicles. PLoS One. 2021;16(5):e0251690. doi: 10.1371/journal.pone.0251690 33989363 PMC8121316

[44] Sarkar MK, Karal MAS, Levadny V, Belaya M, Ahmed M, Ahamed MK, et al. Effects of sugar concentration on the electroporation, size distribution and average size of charged giant unilamellar vesicles. Eur Biophys J. 2022;51(4–5):401–12. doi: 10.1007/s00249-022-01607-y 35716178

[45] Billah MM, Ahmed M, Islam MZ, Yamazaki M. Processes and mechanisms underlying burst of giant unilamellar vesicles induced by antimicrobial peptides and compounds. Biochim Biophys Acta Biomembr. 2024;1866(5):184330. doi: 10.1016/j.bbamem.2024.184330 38679311

[46] Billah MM, Saha SK, Or Rashid MM, Hossain F, Yamazaki M. Effect of osmotic pressure on pore formation in lipid bilayers by the antimicrobial peptide magainin 2. Phys Chem Chem Phys. 2022;24(11):6716–31. doi: 10.1039/d1cp05764b 35234764

[47] Karal MAS, Billah MM, Nasrin T, Moniruzzaman M. Interaction of anionic Fe3O4 nanoparticles with lipid vesicles: a review on deformation and poration under various conditions. RSC Adv. 2024;14(36):25986–6001. doi: 10.1039/d4ra05686h 39161454 PMC11331399

[48] Billah MM, Or Rashid MM, Ahmed M, Yamazaki M. Antimicrobial peptide magainin 2-induced rupture of single giant unilamellar vesicles comprising E. coli polar lipids. Biochim Biophys Acta Biomembr. 2023;1865(3):184112. doi: 10.1016/j.bbamem.2022.184112 36567034

[49] Litster JD. Stability of lipid bilayers and red blood cell membranes. Physics Letters A. 1975;53(3):193–4. doi: 10.1016/0375-9601(75)90402-8

[50] Karal MAS, Levadnyy V, Yamazaki M. Analysis of constant tension-induced rupture of lipid membranes using activation energy. Phys Chem Chem Phys. 2016;18(19):13487–95. doi: 10.1039/c6cp01184e 27125194

[51] Karal MAS, Levadnyy V, Tsuboi T, Belaya M, Yamazaki M. Electrostatic interaction effects on tension-induced pore formation in lipid membranes. Phys Rev E Stat Nonlin Soft Matter Phys. 2015;92(1):012708. doi: 10.1103/PhysRevE.92.012708 26274204

[52] Kulczycki A, Kajdas C. A new attempt to better understand arrehnius equation and its activation energy. Tribol Eng. InTech. 2013. doi: 10.5772/54503

[53] Wadud MA, Karal MAS, Moniruzzaman M, Rashid MMO. Effects of membrane potentials on the electroporation of giant unilamellar vesicles. PLoS One. 2023;18(9):e0291496. doi: 10.1371/journal.pone.0291496 37699026 PMC10497157

[54] Heitz F, Spach G, Seta P, Gavach C. Ion conducting pores induced by oligo-L-alanine. Biochem Biophys Res Commun. 1982;107(2):481–4. doi: 10.1016/0006-291x(82)91516-9 6289832

[55] Hladky SB, Haydon DA. Ion transfer across lipid membranes in the presence of gramicidin A. I. Studies of the unit conductance channel. Biochim Biophys Acta. 1972;274(2):294–312. doi: 10.1016/0005-2736(72)90178-2 5048999

[56] Kelkar DA, Chattopadhyay A. The gramicidin ion channel: a model membrane protein. Biochim Biophys Acta. 2007;1768(9):2011–25. doi: 10.1016/j.bbamem.2007.05.011 17572379

[57] Shillcock JC, Lipowsky R. Tension-induced fusion of bilayer membranes and vesicles. Nat Mater. 2005;4(3):225–8. doi: 10.1038/nmat1333 15711550

[58] Mouritsen OG, Bloom M. Models of lipid-protein interactions in membranes. Annu Rev Biophys Biomol Struct. 1993;22:145–71. doi: 10.1146/annurev.bb.22.060193.001045 8347987

[59] Helfrich W. Elastic properties of lipid bilayers: theory and possible experiments. Z Naturforsch C. 1973;28(11):693–703. doi: 10.1515/znc-1973-11-1209 4273690

[60] Huang HW. Deformation free energy of bilayer membrane and its effect on gramicidin channel lifetime. Biophys J. 1986;50(6):1061–70. doi: 10.1016/S0006-3495(86)83550-0 2432948 PMC1329780

[61] Yang L, Harroun TA, Weiss TM, Ding L, Huang HW. Barrel-stave model or toroidal model? A case study on melittin pores. Biophys J. 2001;81(3):1475–85. doi: 10.1016/S0006-3495(01)75802-X 11509361 PMC1301626

[62] Leontiadou H, Mark AE, Marrink SJ. Molecular dynamics simulations of hydrophilic pores in lipid bilayers. Biophys J. 2004;86(4):2156–64. doi: 10.1016/S0006-3495(04)74275-7 15041656 PMC1304067

[63] Shchelokovskyy P, Tristram-Nagle S, Dimova R. Effect of the HIV-1 fusion peptide on the mechanical properties and leaflet coupling of lipid bilayers. New J Phys. 2011;13:25004. doi: 10.1088/1367-2630/13/2/025004 23505334 PMC3595596

