# Supplementary material for: Electric field-induced rupture kinetics of giant unilamellar vesicles with varying gramicidin A content in membrane
Source: PLoS One. 2026 Jan 5;21(1):e0338817. doi: 10.1371/journal.pone.0338817 (PMC12768252; doi:10.1371/journal.pone.0338817)
Supplement: S1 File — (PDF) [file pone.0338817.s001.pdf]

# Supporting Information (S1)

# Electric field-induced rupture kinetics of giant unilamellar vesicles with varying gramicidin A content in membrane


Md. Tariqul Islam Bhuiyan[1], Mir Jubair Ahamed[1], Rajia Sultana[1,2], Tawfika Nasrin[1], Md. Kabir Ahamed[3], Md. Masum Billah[4]*, and Mohammad Abu Sayem Karal[1]*

[1]Department of Physics, Bangladesh University of Engineering and Technology, Dhaka 1000, Bangladesh
[2]Department of Physics, Bangladesh University of Textiles, Dhaka 1208, Bangladesh
[3]Radiation, Transport and Waste Safety Division, Bangladesh Atomic Energy Regulatory Authority, Agargaon, Dhaka 1207, Bangladesh
[4]Department of Physics, Jashore University of Science and Technology, Jashore 7408, Bangladesh


## S1 Rate constant of rupture for 0.1, 1, and 3% GrA

The time-dependent fraction of intact vesicles ($P_{\text{intact}}$) for 0.1%, 1%, and 3% GrA is shown in S Fig. The experimental data were fitted using the single exponential decay function described by Eq. (4). From these fittings, the rupture rate constants ($k_r$) at $\sigma_e$ = 8 mN/m were determined to be $2.3\times10^{-2}$ $s^{-1}$, $3.2\times10^{-2}$ $s^{-1}$, and $6.8\times10^{-2}$ $s^{-1}$ for 0.1, 1, and 3% GrA, respectively.

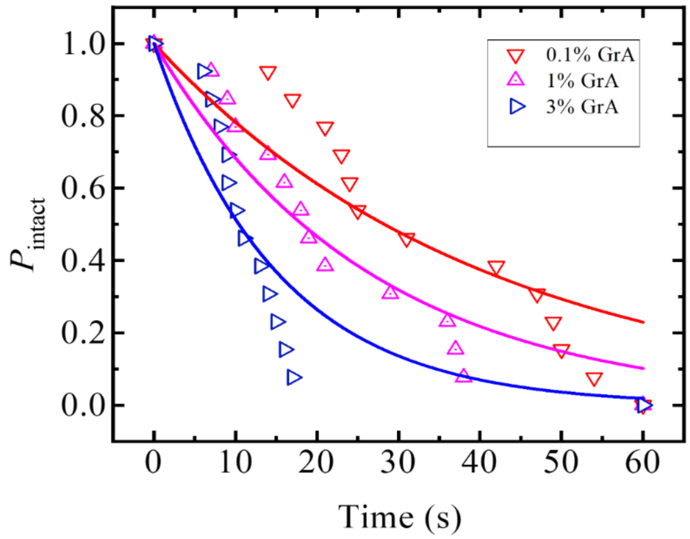


**S1 Fig. Rupture kinetics of DOPG/DOPC/GrA-GUVs at different GrA% under $\sigma_e$ = 8 mN/m.** (A) Time-dependent fraction of intact GUVs ($P_{\text{intact}}$) for 0.1, 1, and 3% GrA, showing the exponential decay behavior. The coefficient of determination ($R^2$) was evaluated for the goodness of fit. The values of $R^2$ were obtained 0.81, 0.91, and 0.83 for 0.1, 1, and 3% GrA, respectively.
